# (2*RS*,4′*RS*)-3′-(3-Chloro-4-meth­oxy­phen­yl)-4′-phenyl-4′*H*-spiro­[indene-2,5′-isoxazol]-1(3*H*)-one ethanol monosolvate

**DOI:** 10.1107/S1600536811032636

**Published:** 2011-08-27

**Authors:** Adil Boughaleb, Hafid Zouihri, Said Gmouh, Abdelali Kerbal, Mohamed El yazidi

**Affiliations:** a Département de Chimie, Faculté des Sciences, Dhar Mehraz, BP 1796 Atlas, 30000 Fés, Morocco; b Laboratoires de Diffraction des Rayons X, Centre Nationale pour la Recherche Scientifique et Technique, Rabat, Morocco; c Centre Nationale pour la Recherche Scientifique et Technique, Rabat, Morocco

## Abstract

The title compound, C_23_H_17_ClN_2_O_3_·C_2_H_6_O, is the stoichiometric 1:1 ethanol solvate of a racemic reaction product, which forms a conglomerate. The refined Flack parameter of 0.36 (3) indicates racemic twinning. In the structure, mol­ecules are linked into zigzag chains by a series of inter­molecular N—H⋯O and O—H⋯O hydrogen bonds.

## Related literature

For general background to dipolar 1,3-cyclo­addition reactions, see: Al Houari *et al.* (2010 ▶); Toth *et al.* (1999 ▶); El yazidi *et al.* (1994 ▶). 
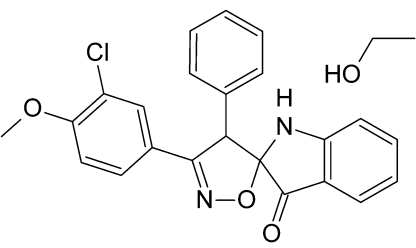


## Experimental

### 

#### Crystal data

C_23_H_17_ClN_2_O_3_·C_2_H_6_O
*M* *_r_* = 450.90
Orthorhombic, 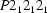

*a* = 8.7112 (2) Å
*b* = 11.3051 (2) Å
*c* = 22.5913 (5) Å
*V* = 2224.81 (8) Å^3^
*Z* = 4
Mo *K*α radiation
μ = 0.21 mm^−1^
*T* = 296 K
0.24 × 0.17 × 0.16 mm

#### Data collection

Bruker APEXII CCD diffractometer
13160 measured reflections
4757 independent reflections
3815 reflections with *I* > 2σ(*I*)
*R*_int_ = 0.027

#### Refinement

*R*[*F* ^2^ > 2σ(*F* ^2^)] = 0.039
*wR*(*F* ^2^) = 0.096
*S* = 1.06
4757 reflections
297 parameters
H atoms treated by a mixture of independent and constrained refinement
Δρ_max_ = 0.16 e Å^−3^
Δρ_min_ = −0.32 e Å^−3^
Absolute structure: Flack (1983 ▶); 1986 Friedel pairs
Flack parameter: 0.36 (3)

### 

Data collection: *APEX2* (Bruker, 2005 ▶); cell refinement: *SAINT* (Bruker, 2005 ▶); data reduction: *SAINT*; program(s) used to solve structure: *SHELXS97* (Sheldrick, 2008 ▶); program(s) used to refine structure: *SHELXL97* (Sheldrick, 2008 ▶); molecular graphics: *PLATON* (Spek, 2009 ▶); software used to prepare material for publication: *publCIF* (Westrip, 2010 ▶).

## Supplementary Material

Crystal structure: contains datablock(s) I, New_Global_Publ_Block. DOI: 10.1107/S1600536811032636/fy2017sup1.cif

Structure factors: contains datablock(s) I. DOI: 10.1107/S1600536811032636/fy2017Isup2.hkl

Supplementary material file. DOI: 10.1107/S1600536811032636/fy2017Isup3.cml

Additional supplementary materials:  crystallographic information; 3D view; checkCIF report

## Figures and Tables

**Table 1:** Hydrogen-bond geometry (Å, °)

*D*—H⋯*A*	*D*—H	H⋯*A*	*D*⋯*A*	*D*—H⋯*A*
N1—H1⋯O4	0.88 (3)	2.12 (3)	2.906 (2)	149 (2)
O4—H4*A*⋯O2^i^	0.82	2.02	2.813 (2)	164

Symmetry code: (i) 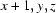.

**Fig. 1.**
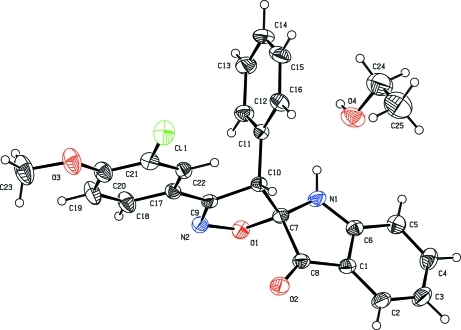
Molecular view of the title compound showing the atom labeling scheme. Displacement ellipsoids are drawn at the 30% probability level. H atoms are represented as small spheres of arbitrary radii.

**Fig. 2.**
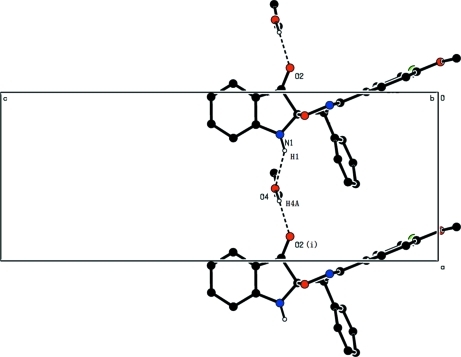
Partial packing view showing the chain formed by N—H···O and O—H···O hydrogen bondings. H atoms not involved in hydrogen bonds have been omitted for clarity. Symmetry codes: (i) x+1, y, z.

## supplementary crystallographic information

### Comment

In general, the 1,3-dipolar cycloaddition of arylnitrile oxides with ethylenic dipolarophiles leads to the regioselective formation of isoxazolines so that the electron withdrawing substituent of the dipolarophile is connected to position 5 of the isoxazoline ring (Al Houari *et al.*, 2010; Toth *et al.*, 1999; El yazidi *et al.*, 1994).

The crystal structure shows that the carbonyl group is bonded to position 5 of the isoxazoline ring, and that the hydrogen atom in position 4 is situated on the same side of the ring as the carbonyl group. The structural study by X-rays is in perfect agreement with the results of IR, ^1^H and ^13^C NMR spectroscopic analysis.

The 1,3-dipolar cycloaddition gave the title compound as a racemic product. However, the crystallization process from ethanol resulted in the formation of a conglomerate in space group *P*2_1_2_1_2_1_.

In the crystal molecules are linked into zigzag chains by a series of intermolecular N—H···O and O—H···O hydrogen-bonding interactions, where both the main and the solvent molecules take part (Table 1).

### Experimental

4 mmol of (2*Z*)-2-benzylidene-1,2-dihydro-3*H*-indol-3-one and 4 mmol of 3-chloro-*N*-hydroxy-4-methoxybenzenecarboximidoyl chloride were dissolved in 30 ml of THF in a 100 ml flask equipped with a condenser. The reaction mixture was refluxed until the complete dissolution of the reagents, and then 1 ml of *N*,*N*-diethylethanamine was added to the reaction. Monitoring the reaction by thin film chromatography revealed the formation of a single cycloaddition product. After filtration, the organic solution was evaporated under reduced pressure. The residue obtained was recrystallized from ethanol.

### Refinement

The H atoms bound to C were treated as riding with their parent atoms [C—H distances are 0.93 Å for CH groups with *U*_iso_(H) = 1.2 *U*_eq_(C), and 0.97 Å for CH_3_ groups with *U*_iso_(H) = 1.5 *U*_eq_(C). The nitrogen- and oxygen-bound H atoms were located in a difference Fourier map. The nitrogen-bound H atom was refined freely, while the H atom of the hydroxyl group was refined as an idealised rotating group and with *U*_iso_(H) = 1.5 *U*_eq_(O).

Refinment with TWIN and BASF instructions in *SHELXL* indicated racemic twinning.

### Crystal data

**Table:**

C_23_H_17_ClN_2_O_3_·C_2_H_6_O	*F*(000) = 944
*M**_r_* = 450.90	*D*_x_ = 1.346 Mg m^−^^3^
Orthorhombic, *P*2_1_2_1_2_1_	Mo *K*α radiation, λ = 0.71073 Å
Hall symbol: P 2ac 2ab	Cell parameters from 265 reflections
*a* = 8.7112 (2) Å	θ = 2.6–26.3°
*b* = 11.3051 (2) Å	µ = 0.21 mm^−^^1^
*c* = 22.5913 (5) Å	*T* = 296 K
*V* = 2224.81 (8) Å^3^	Prism, colourless
*Z* = 4	0.24 × 0.17 × 0.16 mm

### Data collection

**Table:**

Bruker APEXII CCD diffractometer	3815 reflections with *I* > 2σ(*I*)
Radiation source: fine-focus sealed tube	*R*_int_ = 0.027
graphite	θ_max_ = 27.0°, θ_min_ = 1.8°
ω and φ scans	*h* = −10→11
13160 measured reflections	*k* = −14→14
4757 independent reflections	*l* = −28→17

### Refinement

**Table:**

Refinement on *F*^2^	Secondary atom site location: difference Fourier map
Least-squares matrix: full	Hydrogen site location: inferred from neighbouring sites
*R*[*F*^2^ > 2σ(*F*^2^)] = 0.039	H atoms treated by a mixture of independent and constrained refinement
*wR*(*F*^2^) = 0.096	*w* = 1/[σ^2^(*F*_o_^2^) + (0.0469*P*)^2^ + 0.1284*P*] where *P* = (*F*_o_^2^ + 2*F*_c_^2^)/3
*S* = 1.06	(Δ/σ)_max_ < 0.001
4757 reflections	Δρ_max_ = 0.16 e Å^−^^3^
297 parameters	Δρ_min_ = −0.32 e Å^−^^3^
0 restraints	Absolute structure: Flack (1983); 1986 Friedel pairs
Primary atom site location: structure-invariant direct methods	Flack parameter: 0.36 (3)

### Special details

**Table:**

Geometry. All esds (except the esd in the dihedral angle between two l.s. planes) are estimated using the full covariance matrix. The cell esds are taken into account individually in the estimation of esds in distances, angles and torsion angles; correlations between esds in cell parameters are only used when they are defined by crystal symmetry. An approximate (isotropic) treatment of cell esds is used for estimating esds involving l.s. planes.
Refinement. Refinement of F^2^ against ALL reflections. The weighted R-factor wR and goodness of fit S are based on F^2^, conventional R-factors R are based on F, with F set to zero for negative F^2^. The threshold expression of F^2^ > 2sigma(F^2^) is used only for calculating R-factors(gt) etc. and is not relevant to the choice of reflections for refinement. R-factors based on F^2^ are statistically about twice as large as those based on F, and R- factors based on ALL data will be even larger.

### Fractional atomic coordinates and isotropic or equivalent isotropic displacement parameters (Å^2^)

**Table:**

	*x*	*y*	*z*	*U*_iso_*/*U*_eq_	
C1	0.0305 (2)	0.33701 (16)	0.41709 (9)	0.0378 (4)	
C10	0.1211 (2)	0.35087 (15)	0.26128 (8)	0.0333 (4)	
C11	0.2714 (2)	0.39793 (16)	0.23651 (8)	0.0340 (4)	
C12	0.3965 (2)	0.32367 (18)	0.22940 (10)	0.0470 (5)	
C13	0.5324 (3)	0.3680 (2)	0.20606 (11)	0.0590 (6)	
C14	0.5434 (3)	0.4828 (2)	0.18859 (11)	0.0629 (7)	
C15	0.4190 (3)	0.5564 (2)	0.19482 (11)	0.0585 (6)	
C16	0.2839 (2)	0.51488 (18)	0.21932 (10)	0.0471 (5)	
C17	−0.0022 (2)	0.26234 (16)	0.16411 (8)	0.0359 (4)	
C18	−0.0316 (3)	0.16313 (18)	0.12947 (10)	0.0533 (6)	
C19	−0.0905 (3)	0.17326 (19)	0.07365 (11)	0.0613 (7)	
C2	−0.0518 (3)	0.36087 (19)	0.46878 (10)	0.0495 (5)	
C20	−0.1223 (3)	0.28381 (19)	0.04958 (10)	0.0526 (5)	
C21	−0.0928 (2)	0.38300 (17)	0.08457 (9)	0.0423 (5)	
C22	−0.0338 (2)	0.37289 (17)	0.14030 (9)	0.0379 (4)	
C23	−0.2016 (4)	0.2016 (3)	−0.04206 (13)	0.0908 (10)	
C24	0.6087 (4)	0.5038 (3)	0.36539 (16)	0.0920 (9)	
C25	0.4781 (4)	0.5779 (3)	0.37505 (16)	0.1008 (11)	
C3	0.0272 (3)	0.3874 (2)	0.51910 (10)	0.0587 (6)	
C4	0.1875 (3)	0.3900 (2)	0.51872 (10)	0.0569 (6)	
C5	0.2713 (3)	0.36897 (19)	0.46834 (9)	0.0466 (5)	
C6	0.1917 (2)	0.34114 (16)	0.41674 (9)	0.0378 (4)	
C7	0.1305 (2)	0.28684 (16)	0.32140 (8)	0.0362 (4)	
C8	−0.0172 (2)	0.30244 (18)	0.35872 (9)	0.0389 (5)	
C9	0.0618 (2)	0.24995 (15)	0.22427 (8)	0.0330 (4)	
Cl1	−0.12367 (8)	0.52300 (5)	0.05494 (3)	0.06402 (19)	
H1	0.346 (3)	0.324 (2)	0.3510 (10)	0.060 (7)*	
H10	0.0447	0.4144	0.2630	0.040*	
H12	0.3895	0.2445	0.2402	0.056*	
H13	0.6170	0.3184	0.2023	0.071*	
H14	0.6345	0.5113	0.1726	0.075*	
H15	0.4257	0.6348	0.1825	0.070*	
H16	0.2011	0.5659	0.2243	0.056*	
H18	−0.0107	0.0884	0.1446	0.064*	
H19	−0.1096	0.1055	0.0515	0.074*	
H2	−0.1585	0.3587	0.4688	0.059*	
H22	−0.0145	0.4406	0.1625	0.045*	
H23A	−0.1057	0.1606	−0.0465	0.136*	
H23B	−0.2379	0.2263	−0.0802	0.136*	
H23C	−0.2756	0.1497	−0.0242	0.136*	
H24A	0.6892	0.5250	0.3931	0.110*	
H24B	0.6475	0.5166	0.3257	0.110*	
H25A	0.3977	0.5561	0.3481	0.151*	
H25B	0.5062	0.6590	0.3685	0.151*	
H25C	0.4427	0.5684	0.4150	0.151*	
H3	−0.0258	0.4038	0.5539	0.070*	
H4	0.2392	0.4066	0.5538	0.068*	
H4A	0.6442	0.3400	0.3634	0.105*	
H5	0.3779	0.3732	0.4687	0.056*	
N1	0.2497 (2)	0.31383 (17)	0.36182 (8)	0.0444 (4)	
N2	0.07647 (17)	0.14773 (13)	0.24799 (7)	0.0391 (4)	
O1	0.13828 (17)	0.16082 (11)	0.30525 (6)	0.0426 (3)	
O2	−0.14510 (17)	0.28485 (15)	0.33873 (7)	0.0583 (4)	
O3	−0.1799 (2)	0.30291 (14)	−0.00516 (8)	0.0716 (5)	
O4	0.57042 (17)	0.38095 (16)	0.37275 (9)	0.0703 (5)	

### Atomic displacement parameters (Å^2^)

**Table:**

	*U*^11^	*U*^22^	*U*^33^	*U*^12^	*U*^13^	*U*^23^
C1	0.0382 (11)	0.0361 (10)	0.0391 (11)	−0.0006 (8)	0.0064 (9)	0.0047 (8)
C10	0.0287 (9)	0.0337 (9)	0.0374 (10)	0.0030 (8)	0.0024 (8)	0.0008 (7)
C11	0.0332 (11)	0.0352 (9)	0.0336 (10)	−0.0031 (8)	0.0001 (8)	−0.0006 (8)
C12	0.0396 (12)	0.0441 (11)	0.0571 (13)	−0.0002 (9)	0.0087 (10)	−0.0001 (9)
C13	0.0373 (12)	0.0655 (15)	0.0741 (17)	−0.0016 (11)	0.0164 (12)	−0.0106 (13)
C14	0.0480 (14)	0.0782 (18)	0.0625 (16)	−0.0275 (13)	0.0121 (12)	0.0003 (13)
C15	0.0599 (16)	0.0500 (13)	0.0657 (16)	−0.0216 (11)	0.0001 (13)	0.0119 (11)
C16	0.0464 (12)	0.0387 (10)	0.0561 (13)	−0.0035 (9)	−0.0030 (11)	0.0045 (10)
C17	0.0311 (10)	0.0377 (10)	0.0390 (11)	−0.0025 (8)	−0.0014 (8)	−0.0013 (8)
C18	0.0685 (15)	0.0365 (11)	0.0549 (14)	−0.0078 (10)	−0.0150 (12)	0.0033 (10)
C19	0.0843 (19)	0.0429 (12)	0.0566 (15)	−0.0146 (11)	−0.0225 (13)	−0.0047 (10)
C2	0.0509 (13)	0.0523 (13)	0.0454 (12)	−0.0015 (10)	0.0150 (11)	0.0028 (10)
C20	0.0555 (13)	0.0545 (13)	0.0476 (13)	−0.0071 (11)	−0.0158 (12)	0.0031 (10)
C21	0.0380 (12)	0.0417 (10)	0.0472 (12)	0.0030 (8)	−0.0033 (10)	0.0048 (9)
C22	0.0344 (10)	0.0375 (10)	0.0417 (11)	0.0010 (8)	−0.0001 (9)	−0.0022 (8)
C23	0.129 (3)	0.0773 (18)	0.0661 (19)	−0.0205 (18)	−0.0483 (19)	−0.0026 (15)
C24	0.078 (2)	0.090 (2)	0.108 (3)	−0.0139 (19)	0.0090 (19)	−0.0018 (18)
C25	0.106 (3)	0.074 (2)	0.122 (3)	−0.0016 (19)	−0.005 (2)	0.0111 (19)
C3	0.0709 (18)	0.0657 (15)	0.0396 (13)	0.0004 (13)	0.0141 (12)	0.0017 (11)
C4	0.0744 (18)	0.0605 (14)	0.0357 (12)	0.0038 (12)	−0.0051 (11)	0.0001 (10)
C5	0.0484 (13)	0.0502 (12)	0.0413 (12)	0.0023 (10)	−0.0078 (10)	0.0075 (9)
C6	0.0410 (11)	0.0333 (9)	0.0392 (11)	0.0020 (8)	0.0030 (9)	0.0054 (8)
C7	0.0304 (10)	0.0396 (10)	0.0387 (10)	0.0008 (8)	0.0041 (9)	−0.0017 (8)
C8	0.0334 (12)	0.0391 (10)	0.0442 (12)	−0.0022 (8)	0.0066 (9)	0.0003 (9)
C9	0.0265 (9)	0.0351 (9)	0.0374 (10)	−0.0009 (7)	0.0052 (8)	0.0013 (8)
Cl1	0.0852 (4)	0.0498 (3)	0.0571 (3)	0.0136 (3)	−0.0127 (4)	0.0081 (3)
N1	0.0296 (10)	0.0642 (11)	0.0393 (10)	−0.0021 (8)	0.0047 (8)	−0.0020 (8)
N2	0.0414 (10)	0.0371 (9)	0.0389 (9)	−0.0005 (7)	0.0019 (7)	0.0001 (7)
O1	0.0502 (8)	0.0370 (7)	0.0405 (8)	0.0051 (6)	0.0008 (7)	0.0041 (6)
O2	0.0312 (9)	0.0843 (11)	0.0595 (10)	−0.0069 (7)	0.0045 (7)	−0.0158 (8)
O3	0.0952 (14)	0.0641 (10)	0.0555 (10)	−0.0077 (9)	−0.0353 (10)	−0.0004 (8)
O4	0.0398 (9)	0.0696 (12)	0.1014 (14)	−0.0027 (8)	0.0102 (9)	−0.0073 (10)

### Geometric parameters (Å, °)

**Table:**

C1—C2	1.397 (3)	C23—H23C	0.9600
C10—H10	0.9800	C23—H23B	0.9600
C10—C7	1.541 (3)	C23—H23A	0.9600
C10—C11	1.520 (2)	C24—H24B	0.9700
C10—C9	1.506 (2)	C24—H24A	0.9700
C11—C12	1.385 (3)	C24—C25	1.429 (5)
C11—C16	1.382 (3)	C25—H25C	0.9600
C12—H12	0.9300	C25—H25B	0.9600
C12—C13	1.389 (3)	C25—H25A	0.9600
C13—H13	0.9300	C3—H3	0.9300
C13—C14	1.361 (4)	C4—H4	0.9300
C14—H14	0.9300	C4—C3	1.396 (4)
C15—H15	0.9300	C4—C5	1.373 (3)
C15—C14	1.373 (4)	C5—H5	0.9300
C16—H16	0.9300	C6—C1	1.405 (3)
C16—C15	1.383 (3)	C6—C5	1.392 (3)
C17—C9	1.476 (3)	C8—C7	1.549 (3)
C17—C18	1.391 (3)	C8—C1	1.437 (3)
C18—H18	0.9300	N1—H1	0.88 (3)
C18—C19	1.366 (3)	N1—C7	1.416 (3)
C19—H19	0.9300	N1—C6	1.375 (3)
C2—H2	0.9300	N2—O1	1.409 (2)
C2—C3	1.362 (3)	N2—C9	1.280 (2)
C20—C19	1.391 (3)	O1—C7	1.472 (2)
C21—Cl1	1.739 (2)	O2—C8	1.218 (2)
C21—C20	1.396 (3)	O3—C23	1.429 (3)
C22—H22	0.9300	O3—C20	1.352 (3)
C22—C17	1.388 (3)	O4—H4A	0.8200
C22—C21	1.365 (3)	O4—C24	1.438 (4)
			
C6—C1—C8	107.05 (17)	H23A—C23—H23C	109.5
C2—C1—C8	132.17 (19)	O3—C23—H23C	109.5
C2—C1—C6	120.7 (2)	H23A—C23—H23B	109.5
C7—C10—H10	110.1	O3—C23—H23B	109.5
C11—C10—H10	110.1	O3—C23—H23A	109.5
C9—C10—H10	110.1	H24A—C24—H24B	108.0
C11—C10—C7	116.32 (16)	O4—C24—H24B	109.4
C9—C10—C7	98.72 (13)	C25—C24—H24B	109.4
C9—C10—C11	110.86 (15)	O4—C24—H24A	109.4
C12—C11—C10	120.54 (16)	C25—C24—H24A	109.4
C16—C11—C10	120.42 (17)	C25—C24—O4	111.3 (3)
C16—C11—C12	119.01 (17)	H25B—C25—H25C	109.5
C13—C12—H12	120.1	H25A—C25—H25C	109.5
C11—C12—H12	120.1	C24—C25—H25C	109.5
C11—C12—C13	119.73 (19)	H25A—C25—H25B	109.5
C12—C13—H13	119.5	C24—C25—H25B	109.5
C14—C13—H13	119.5	C24—C25—H25A	109.5
C14—C13—C12	120.9 (2)	C4—C3—H3	119.8
C15—C14—H14	120.2	C2—C3—H3	119.8
C13—C14—H14	120.2	C2—C3—C4	120.3 (2)
C13—C14—C15	119.5 (2)	C3—C4—H4	118.9
C16—C15—H15	119.8	C5—C4—H4	118.9
C14—C15—H15	119.8	C5—C4—C3	122.2 (2)
C14—C15—C16	120.4 (2)	C6—C5—H5	121.0
C15—C16—H16	119.8	C4—C5—H5	121.0
C11—C16—H16	119.8	C4—C5—C6	117.9 (2)
C11—C16—C15	120.3 (2)	C5—C6—C1	120.04 (19)
C18—C17—C9	120.73 (16)	N1—C6—C1	111.39 (18)
C22—C17—C9	121.14 (16)	N1—C6—C5	128.57 (18)
C22—C17—C18	118.13 (17)	C10—C7—C8	112.47 (16)
C17—C18—H18	119.3	O1—C7—C8	106.46 (14)
C19—C18—H18	119.3	N1—C7—C8	103.50 (15)
C19—C18—C17	121.38 (19)	O1—C7—C10	103.80 (14)
C20—C19—H19	119.6	N1—C7—C10	120.40 (16)
C18—C19—H19	119.6	N1—C7—O1	109.55 (15)
C18—C19—C20	120.74 (19)	C1—C8—C7	106.86 (16)
C1—C2—H2	120.6	O2—C8—C7	122.64 (18)
C3—C2—H2	120.6	O2—C8—C1	130.48 (19)
C3—C2—C1	118.7 (2)	C17—C9—C10	124.70 (15)
C19—C20—C21	117.63 (19)	N2—C9—C10	114.66 (16)
O3—C20—C21	117.26 (19)	N2—C9—C17	120.58 (16)
O3—C20—C19	125.11 (19)	C7—N1—H1	123.1 (15)
C20—C21—Cl1	118.99 (16)	C6—N1—H1	124.9 (15)
C22—C21—Cl1	119.30 (15)	C6—N1—C7	111.18 (17)
C22—C21—C20	121.64 (18)	C9—N2—O1	109.12 (14)
C17—C22—H22	119.8	N2—O1—C7	108.16 (12)
C21—C22—H22	119.8	C20—O3—C23	117.03 (19)
C21—C22—C17	120.49 (18)	C24—O4—H4A	109.5
H23B—C23—H23C	109.5		

### Hydrogen-bond geometry (Å, °)

**Table:**

*D*—H···*A*	*D*—H	H···*A*	*D*···*A*	*D*—H···*A*
N1—H1···O4	0.88 (3)	2.12 (3)	2.906 (2)	149 (2)
O4—H4A···O2^i^	0.82	2.02	2.813 (2)	164.

Symmetry codes: (i) *x*+1, *y*, *z*.

## References

[bb1] Al Houari, G., Bennani, A. K., Bennani, B., Daoudi, M., Benlarbi, N., El Yazidi, M., Garrigues, B. & Kerbal, A. (2010). *J. Maroc. Chim. Heterocycl.* **9**, 36–43.

[bb2] Bruker (2005). *APEX2* and *SAINT* Bruker AXS Inc., Madison, Wisconsin, USA.

[bb3] El yazidi, M., Daou, B., Bougrin, K. & Soufiaoui, M. (1994). *J. Soc. Maroc. Chim.* **3**, 54–51.

[bb4] Flack, H. D. (1983). *Acta Cryst.* A**39**, 876–881.

[bb5] Sheldrick, G. M. (2008). *Acta Cryst.* A**64**, 112–122. 10.1107/S0108767307043930 18156677

[bb6] Spek, A. L. (2009). *Acta Cryst.* D**65**, 148–155. 10.1107/S090744490804362X PMC2631630 19171970

[bb7] Toth, G., Balazs, B., Levai, A., Fisera, L. & Jedlovska, E. (1999). *J. Mol. Struct.* **508**, 29–36.

[bb8] Westrip, S. P. (2010). *J. Appl. Cryst.* **43**, 920–925.

